# Prevalence and Incidence of Non-neutralizing Antibodies in Congenital Hemophilia A— A Systematic Review and Meta-Analysis

**DOI:** 10.3389/fimmu.2020.00563

**Published:** 2020-05-07

**Authors:** A. Abdi, M. R. Bordbar, S. Hassan, F. R. Rosendaal, J. G. van der Bom, J. Voorberg, K. Fijnvandraat, S. C. Gouw

**Affiliations:** ^1^Department of Pediatric Hematology, Amsterdam UMC, Emma Children's Hospital, University of Amsterdam, Amsterdam, Netherlands; ^2^Hematology Research Center, Nemazee Hospital, Shiraz University of Medical Sciences, Shiraz, Iran; ^3^Department of Clinical Epidemiology, Leiden University Medical Center, Leiden, Netherlands; ^4^Center for Clinical Transfusion Research, Sanquin Research, Leiden, Netherlands; ^5^Department of Molecular Cellular Hemostasis, Sanquin Research and Landsteiner Laboratory, Amsterdam, Netherlands; ^6^Department of Experimental Vascular Medicine, Amsterdam University Medical Center, University of Amsterdam, Amsterdam, Netherlands

**Keywords:** hemophilia, FVIII, FIX, non-neutralizing antibodies, anti-drug antibodies, ADA assay, inhibitors

## Abstract

**Objectives:** In hemophilia A the presence of non-neutralizing antibodies (NNAs) against Factor VIII (FVIII) may predict the development of neutralizing antibodies (inhibitors) and accelerate the clearance of administrated FVIII concentrates. This systematic review aimed to assess: (1) the prevalence and incidence of NNAs in patients with congenital hemophilia without inhibitors and (2) the association between NNAs and patient and treatment characteristics.

**Methods:** We conducted a search in MEDLINE, Embase, Web of Science and the Cochrane database. We included cross-sectional and longitudinal studies reporting on NNAs in patients with hemophilia A and B, who were inhibitor-negative at the start of the observation period. Data were extracted on: hemophilia type and severity, patient and treatment characteristics, NNA prevalence and incidence, NNA assays and inhibitor development. Two independent reviewers performed study selection, data extraction and risk of bias assessment, using adapted criteria of the Joanna Briggs Institute. Studies were classified as high-quality when ≥5/9 criteria were met. NNA assays were classified as high-quality when both quality criteria were met: (1) use of positive controls and (2) competition with FVIII to establish FVIII-specificity. We reported NNA prevalence and incidence for each study. The pooled NNA prevalence was assessed for well-designed studies in previously treated patients, employing high-quality NNA assays.

**Results:** We included data from 2,723 inhibitor-negative patients with hemophilia A, derived from 28 studies. Most studies were cross-sectional (19/28) and none reported on NNAs in hemophilia B. Study design was of high quality in 16/28 studies and the NNA assay quality was high in 9/28 studies. Various NNA assays were used, predominantly ELISA (18/28) with different cut-off values. We found a large variety in NNA prevalence (Range, 0–100%). The pooled NNA prevalence in high-quality studies was 25% (95% CI, 16–38%). The incidence of new NNA development was reported in one study (0.01 NNA per person-exposure day).

**Conclusion:** This systematic review identified studies that were heterogeneous in study design, patient population and NNA assay type, with NNA prevalence ranging from 0 to 100% in inhibitor-negative patients with hemophilia A. The pooled NNA prevalence was 25% in high-quality studies including only previously treated patients and performing high-quality NNA assays.

## Introduction

The development of neutralizing antibodies (inhibitors) against Factor VIII (FVIII) or Factor IX (FIX) is a major complication of the treatment of hemophilia patients with clotting factor concentrates. Inhibitors impair the pro-coagulant effect of FVIII or FIX concentrates, rendering replacement therapy ineffective and increasing the susceptibility to major bleeding episodes (1). It is estimated that about 30% of patients with severe and 13% of patients with non-severe hemophilia A develop an inhibitor during the treatment course (2–4). Inhibitor prevalence in hemophilia B has been reported to be 1.5–3% overall and 9–23% in severe patients (5, 6). Therefore, inhibitor development is associated with considerable morbidity and mortality (2, 7, 8).

Previous studies report that non-neutralizing antibodies (NNAs) against FVIII may also be detected in a considerable number of patients with hemophilia A, as well as in healthy individuals (9–14). NNAs are usually of the immunoglobulin G (IgG) isotype, frequently directed toward the heavy-chain and especially the B-domain of FVIII (9, 10, 15). NNAs of the IgM and IgA isotype have also been reported in recent studies (9, 10, 16).

The significance of NNAs is not well-understood. It has been suggested that these antibodies are a predictor for future inhibitor development (17, 18). Furthermore, NNAs may also increase the clearance of administrated FVIII concentrate from the circulation, thereby reducing the plasma concentration of FVIII and limiting effective hemostasis to control bleeding (15, 19). In a study among 42 patients with severe and moderate hemophilia A, the presence of high-titer FVIII-specific NNAs was associated with reduced FVIII half-life in comparison to patients without NNAs (median 7.8 h, IQR 6.6–9.2 vs. 10.4 h, IQR 8.9–13.8) (20).

Whereas, the prevalence of inhibitors is well-known, this is less precisely defined for NNAs. In contrast with inhibitors that are measured by standardized assays (Bethesda or Nijmegen-modified Bethesda assay), there is no standardized assay to detect NNAs (21, 22). Consequently, a variety of laboratory methods are used (10, 13, 23). In addition to other differences in study design and patient populations, this contributes to the widely varying reports of NNA prevalence.

In this systematic review we aimed: (1) to obtain more precise estimates of the prevalence and incidence of NNAs in patients with congenital hemophilia without inhibitors and (2) to assess the association between the presence of NNAs and patient and treatment characteristics.

## Materials and Methods

This systematic review is reported in accordance with the Preferred Reporting Items for Systematic Reviews and Meta-Analysis (PRISMA) (www.prisma-statement.org) (24). The inclusion criteria and the methodological quality criteria were specified and documented in a protocol in advance.

### Study Eligibility Criteria

#### Studies

Cross-sectional or longitudinal studies reporting the prevalence or incidence of NNAs in congenital hemophilia, published as an article or letter in a peer-reviewed journal, were eligible for inclusion, without restriction on publication date or language. Studies not clearly reporting the method employed to measure NNAs and studies including fewer than 10 patients, were excluded.

#### Patients

Eligible for inclusion were patients with congenital hemophilia A or B who were inhibitor-negative at the start of the study observation period, regardless of previous clotting factor treatment. Patients that received previous treatment with clotting factor concentrate, were defined as previously treated patients, regardless of the cumulative number of exposure days. Patients that had not yet received any previous treatment with clotting factor concentrate at study entry, were defined as previously untreated patients. Absence of an inhibitor needed to be confirmed with a Bethesda assay, according to the cut-off value used by the investigators of the original studies.

#### Endpoints

The primary endpoints were the prevalence and incidence of NNAs. The secondary endpoints were the prevalence and incidence of NNAs, stratified by immunoglobulin (Ig) isotype and IgG subclass. The presence of NNAs was defined as having a positive antibody titer according to the NNA assay (Anti-Drug Antibody assay) and the cut-off value used by the original publication, in patients who were inhibitor-negative based on a Bethesda assay (25).

### Search

Studies were identified by searching the following electronic databases: MEDLINE, Embase, Web of Science and the Cochrane database. The reference lists of the retrieved publications were searched to identify additional relevant publications. We used the following search terms to search all databases: hemophilia A, factor VIII, factor 8, hemophilia B, factor 9, factor IX, non-neutralizing, antibodies, neutralizing. The full search is listed in Supplementary Data 1. The search was designed and supervised by an experienced librarian. The first search was conducted on July 12, 2018. An update of the search in MEDLINE was run on September 11, 2019.

### Study Selection

Two of the authors (AA and MB) screened the titles and abstracts independently to select relevant articles. The full-text of selected articles were reviewed to assess their eligibility for inclusion. In case of any doubt for eligibility or disagreement between the reviewers, this was discussed with a methodological expert (SG).

### Data Collection Process

We excluded duplicate studies by checking the authors' names, authors' affiliations and catchment areas. When studies included overlapping patient cohorts, assessed during the same time period, we included the study containing the highest number of patients. Studies that included 2 or more cohorts were included, when data extraction was possible for each cohort.

### Data Items

The following data were extracted from each included study: study characteristics (i.e., year of publication, study period, study design), population characteristics (i.e., number of inhibitor-negative patients, hemophilia type, hemophilia severity), patient characteristics (i.e., treatment history, inhibitor development), laboratory characteristics (type of NNA and inhibitor assay and cut-off values for positivity) and the prevalence and incidence of NNAs (overall and for each Ig class and IgG subclass).

### Quality Assessment

Critical appraisal of studies was assessed by two reviewers independently (AA and MB). The Joanna Briggs Institute (JBI) checklist for prevalence studies was adapted and used to assess the methodological quality of each included study (Supplementary Data 2) (26). Using the formula provided by the JBI guideline, a sample size of ≥139 was considered adequate. Studies were classified as high-quality when at least 5 of the 9 criteria of the adapted JBI checklist were met.

In compliance with the most recent regulatory guideline, we defined two criteria to assess the quality of the various laboratory methods used to detect NNAs: (1) the use of positive controls as an internal standard and (2) the measurement of FVIII-specificity by means of a competition assay (27). NNA assays were classified as high-quality, when they met both of the quality criteria. The quality assessment of NNA assays, was included into the JBI checklist (Supplementary Data 2, question 6).

### Data Synthesis

The patient and treatment characteristics were described using median and interquartile range (IQR) or range (R) for continues variables and count and percentage for categorical variables. Exact 95% Confidence Intervals (95% CI) of the reported prevalence and incidence rates were calculated by means of the Wilson method, using an online tool for the analysis of epidemiologic data (http://epitools.ausvet.com.au).

For cross-sectional studies, in inhibitor-negative patients, the prevalence of NNAs was determined by calculating the proportion of the number of NNA-positive patients of the total number of patients. For longitudinal studies, the prevalence was calculated using the patient numbers at the end of follow-up.

Depending on the way it was reported in the original study, we reported the incidence of NNAs as the cumulative incidence (the proportion of cases in a given time-period) or as the incidence rate (the rate of new cases per person-exposure day). The association between NNA status and subsequent inhibitor development was assessed by calculating the incidence rate ratio of inhibitor formation in NNA-positive patients, compared to NNA-negative patients for each study.

### Meta-Analysis of NNA Prevalence

We pooled the prevalence of NNAs in the studies including only previously treated patients and employing high-quality NNA assays. In advance, we hypothesized that NNA incidence and prevalence differs between previously treated patients and previously untreated patients. Therefore, in order to provide a meaningful estimate of NNA prevalence, we pooled the data of studies including only previously treated patients.

Because conventional methods for meta-analysis can be biased when the outcome NNA prevalence is rare and when continuity corrections are used, we applied the Binomial-Normal model for the meta-analysis of NNA prevalence (28, 29). We explored heterogeneity by estimating the between-study variance (τ^2^) and by visually assessing the extent to which the 95% CIs of the individual studies overlapped. The meta-analysis was performed in R (version 3.6.1), using the metafor package (28, 30).

In these same studies, we also investigated whether NNA prevalence differed according to severity of disease and inhibitor history. When appropriate, meta-regression analysis was performed.

### Data Evaluation

#### Small Study Data Trends

To evaluate whether small study data trends were present, all studies were sorted in a forest plot, according to sample size and asymmetry of the forest plots was visually assessed (31).

## Results

### Study Selection

**Figure 1:**
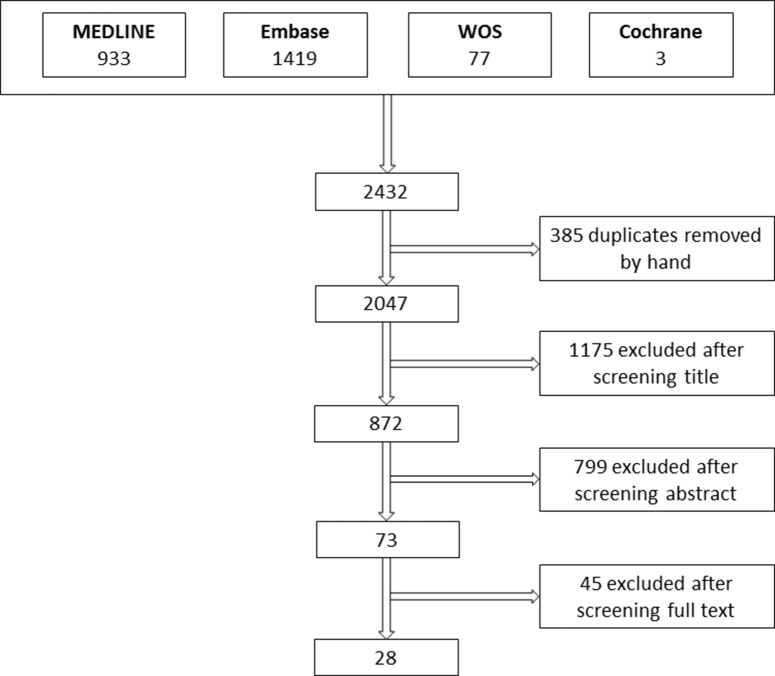
Flow chart of study selection. WOS, Web of Science.

The flow chart of the study selection process is presented in Figure 1. Using the above search strategy, we identified a total of 2,047 unique articles. After title and abstract screening, 73 articles were identified as being potentially relevant. After full text reading and application of the inclusion criteria, 28 studies were eligible for inclusion. The reasons for exclusion after full-text screening were: small sample size (*n* = 4), duplicate publication of results (*n* = 2), unclear methods or insufficient data (*n* = 7), or not meeting the inclusion criteria (*n* = 32). Supplementary Table 1 summarizes the studies that appeared to meet eligibility criteria but on further inspection did not.

### Study and Patient Characteristics

The study and patient characteristics are summarized in Table 1. Studies were all published in English, between 1994 and 2019. Seventeen studies were (partly) conducted in Europe and the majority had a cross-sectional design (19/28). The studies included a total of 3,208 patients with congenital hemophilia A, including 2,723 inhibitor-negative patients. In 14 studies, data on inhibitor history were available, involving 1,583 inhibitor-negative patients, of whom 118 had had an inhibitor in the past. The majority of patients were adult previously treated patients, with severe hemophilia A. In eight of the 11 studies that included information on FVIII product-type, recombinant FVIII (rFVIII) was the most used product. There were no studies with information on NNA prevalence or incidence in patients with hemophilia B. Nor did the cohorts of excluded articles provide information on patients with hemophilia B.

**Table 1:** Study and patient characteristics.

**Source**	**Country**	**Design**	**Included study population**	***N* total**	***N* Inhibitor negative**	**Past inhibitor *n* (%)**	**Severity**			**Age Median (IQR/R)**	**Previous Treatment Cum EDs**	**FVIII product type in >50% of patients**
							**Severe**	**Mod**	**Mild**			
**ELISA**												
David et al. (32)	India	CS	Severe HA PTPs, with and without inhibitor.	312	252	NR	252	0	0	NR	>5	NR
Cannavo et al. (18)	International^a^	LT	Severe HA PUPs <6 Y.	237	237	0	237	0	0	13 M (R 0–67)	0^h^	pFVIII
Gangadharan et al. (17)*	International^b^	LT	Severe HA PUPs.	25	15	0	15	0	0	NR	0	rFVIII (all)
Hofbauer et al. (20)	Austria	CS	Severe and moderate HA PTPs, without current or past inhibitor.	42	42	0	37	5	0	31 Y (R 18–61, IQR 24–44)	NR (PTPs)	rFVIII
Hofbauer et al. (10)	Austria, Germany, Poland	CS	Severe PTPs, with and without inhibitor (no past inhibitor). HS and AHA patients.	101	77	0	77	0	0	36 Y (IQR 26–43)^f^	≥100	NR
Klintman et al. (33)	Sweden^c^	CS	Severe HA PUPs and PTPs without current inhibitor	259	201	79 (39)	201	0	0	NR	NR (PUPs and PTPs)	NR
Klintman et al. (34)	Sweden	LT	Severe and moderate HA PTPs on prophylaxis, without current inhibitor. Brother pairs.	130	78	4 (5)	74	4	0	25.5 Y (R 1–68)	NR (PTPs)	rFVIII
Whelan et al. (9)	Austria, Germany, Poland	CS	Severe HA PTPs, with and without inhibitor (2 groups without inhibitor: after succesful ITI and without inhibitor in past).	120	100	23 (23)	100	0	0	NR	≥100	NR
Moore et al. (35)*	UK	CS	HA, without inhibitor and AHA patients.	46	46	NR	NR	NR	NR	NR	NR	NR
Lillicrap et al. (36)*	Canada	LT	HA, all severities, with and without inhibitor.	392	368	NR	NR	NR	NR	NR	NR	NR
Vincent et al. (37)	Canada	CS	HA PTPs, with and without inhibitor, HS and AHA.	60	50	1 (2)	NR	NR	NR	NR	NR (PTPs)	rFVIII (all)
Towfighi et al. (16)	Iran	CS	Severe HA PTPs with inhibitor, HA PTPs of all severities without inhibitor and HS.	60	30	NR	23	4	3	12-58 Y^g^	NR (PTPs)^i^	NR
Ling et al. (38)	Australia	CS	HA, all severities, with and without inhibitor and AHA patients.	45	26	NR	NR	NR	NR	NR	NR (PTPs)	pFVIII (all)
Shetty et al. (39)	USA	CS	HA, all severities, with and without inhibitor and HS.	312	288	1 (0)	NR	NR	NR	NR	NR (PTPs)	NR
Vianello et al. (40)	Italy	CS	Severe HA PTPs, with and without inhibitor, without FVIII infusion in past month.	33	26	NR	26	0	0	31.5 (IQR 25–39; R 15–54)	NR (PTPs)	pFVIII (all)
Batlle et al. (11)	Spain	CS	HA PTPs, all severities, with and without inhibitor and HS.	124	112	6 (5)	59	28	25	24.4 Y (R 2–78)	NR (PTPs)	NR
Dazzi et al. (12)	Italy	CS	HA PTPs, all severities, without inhibitor.^e^	23	22	1 (5)	8	6	8	NR	NR (PTPs)	NR
Mondorf et al. (41)	Germany	CS	HA, all severities, with and without inhibitor.	53	46	3 (7)	NR	NR	NR	NR	NR	NR
**FLUORESCENCE BASED ASSAY**												
Boylan et al. (42)	USA	LT	HA PTPs, with and without inhibitor and HS.	371	295	0	NR	NR	NR	NR	NR (PTPs)	NR
Butenas et al. (43)	Canada	CS	Severe HA PTPs, with and without inhibitor	34	18	NR	18	0	0	6 Y (IQR 4–30; R 1–39	NR (PTPs)	rFVIII
Zakarija et al. (44)	USA	CS	HA PTPs, all severities, with and without inhibitor.	46	44	NR	31	3	10	39 Y (R 18–86; IQR 32–48)	NR (PTPs)	rFVIII
Krudysz-Amblo (13)	Canada, USA and Poland	CS	HA, all severities, with and without inhibitor and HS.	39	39	NR	18	4	10	NR	NR	NR
**X-MAP**												
Clere et al. (45)	France	LT	HA PTPs, all severities, without inhibitor.	12	12	NR	7	2	3	NR	NR (PTPs)	rFVIII
Lebreton et al. (15)	France	CS	HA PTPs, without inhibitor.	210	210	NR	144	34	32	26 Y (R 1–85)	NR (PTPs)^j^	rFVIII
**IMMUNOPRECIPATION**												
Klinge et al. (23)	Germany	LT	HA PTPs, all severities, with and without inhibitor.	40	20	0	8	9	3	NR	NR (PTPs)	NR
Scandella et al. (46)	International^d^	LT	HA PUPs, all severities.	NR	36	NR	36	0	0	NR	NR (PUPs)	NR
**FLOW CYTOMETRY**												
Irigoyen et al. (47)	Argentina	LT	Severe HA PTPs, with and without inhibitor, at least 7 days after last FVIII infusion.	30	17	NR	17	0	0	NR	NR (PTPs)	NR
**NAME OF NNA ASSAY NOT REPORTED**												
Shurafa and Kithier (14)	USA	CS	HA, without inhibitor.	16	16	NR	NR	NR	NR	NR	NR	NR

Studies are ordered according to NNA assay type and publication date. IQR, interquartile range; R, range; M, months; Y, years; EDs, exposure days; CS, cross-sectional; LT, longitudinal; NR, not reported; PTPs, previously treated patients; PUPs, previously untreated patients; HA, hemophilia A; HS, healthy subjects; AHA, acquired hemophilia A; rFVIII, recombinant FVIII; pFVIII, plasma FVIII. *Conference abstracts, no full-text available.

a *Centers participating in the Survey of Inhibitors in Plasma-Product Exposed Toddlers (SIPPIT) study: India, Egypt, Iran, United States, Italy, and other countries (48)*.

b *Countries were not reported in abstract, but the analyzed samples were from the Hemophilia Inhibitor PUP (HIP) study, that was performed in multiple centers globally*.

c *Study included patients from two cohorts: The Malmo International Brother Study (MIBS) and Hemophilia Inhibitor Genetics Study (HIGS) (49, 50)*.

d *Study included severe PUPs from two multicenter studies: a study that assessed the safety of recombinant FVIII (RECOMBINATE) and a study that compared the safety of recombinant vs. plasma FVIII, performed by the pediatric group of the German Society on Thrombosis and Haemostasis (GTH). The study was still ongoing and the final data were published in 2002 (51, 52)*.

e *One patient was a female carrier with a baseline FVIII activity of 25%, probably due to an extreme lionization*.

f *Median age was only reported for patients with high-titer NNAs (n = 4), defined as NNA titer > 1:80*.

g *Median age was not reported. Not reported whether these values represent the range or interquartile range*.

h *All patients were PUPs or minimally exposed (< 5 times) to blood components (whole blood, fresh-frozen plasma, packed red cells, platelets, or cryoprecipitate)*.

i *All patients were treated with prothrombin complex or human recombinant FVIII*.

j *Treatment was started at least 1 year before blood sampling, without recent switch between rFVIII or pFVIII*.

### NNA and Inhibitor Assay Characteristics

The characteristics of the NNA and inhibitor assays are provided in Table 2, including the results of the quality assessment of the NNA assays. An ELISA was used in 18 of 28 studies. Other studies employed fluorescence based assay (FLI, *n* = 4), multiplexed assay (X-MAP, *n* = 2), immunoprecipitation (IP, *n* = 2), and flow cytometry (FC, *n* = 1). In one study, the NNA assay was not reported (14). Finally, in one study FC and ELISA were compared. As the focus of this study was on the FC NNA detection method, the ELISA assay was not further described (47). A wide range of cut-off values for NNA-positivity was used, generally (12/28 studies) based on healthy controls (+2SD, +3SD). Four studies quantified the FVIII-binding affinity of detected NNAs, measured by ELISA (*n* = 3) or IP (*n* = 1) (17, 20, 46).

**Table 2:** NNA assay and inhibitor assay characteristics.

**Source**	**NNA assay characteristics**						**Inhibitor assay characteristics**	
	**Assay type**	**Cut-off**	**Affinity measured**	**Quality assessment**			**Assay type**	**Cut-off (BU/mL)**
				**Positive control**	**FVIII specificity**	**Quality**		
David et al. (32)	ELISA	OD 490 nm>0.136 or >2SD above mean OD of HC^d^	No	–	–		NBA	0.6
Cannavo et al. (53)	ELISA	OD 492 nm>1.64 μg/mL^e^	No	+	–		mNBA	0.4
Gangadharan et al. (17)	ELISA	titer≥1:20^f^	Yes	+	+		NBA	0.6
Hofbauer et al. (20)	ELISA	titer≥1:20^f^	Yes	+	+		NBA	0.4
Hofbauer et al. (10)	ELISA	titer≥1:20^f^	Yes	+	+		NBA	1.0
Klintman et al. (33)	ELISA^a^	OD>3SD above mean OD of HC^d^	No	+	+		NBA and BA	0.9 and 0.6
Klintman et al. (34)	ELISA^a^	OD>3SD above mean OD of HC^d^	No	+	+		NBA	0.4
Whelan et al. (9)	ELISA	titer≥1:20^f^	No	+	+		NBA	1.0
Moore et al. (35)	ELISA	OD > manufacturer's kit control preparation^g^	No	NR	NR		BA	NR
Lillicrap et al. (36)	ELISA^a^	OD>3SD above mean OD of HC^d^	No	+	NR		NBA and BA	0.6
Vincent et al. (37)	ELISA^a^	OD>3SD above mean OD of HC^d^	No	+	–		mNBA	0.6
Towfighi et al. (16)	ELISA	OD (492 nm)>2SD above mean OD of HC^d^	No	–	–		mBA	1.0
Ling et al. (38)	ELISA^a^	OD>3SD above mean OD of HC^d^	No	+	–		NBA	0.5
Shetty et al. (39)	ELISA	NR	No	–	–		NBA	NR
Vianello et al. (40)	ELISA	OD (450 nm)>3SD above mean OD of three blanks^d^	No	–	+		BA	NR
Batlle et al. (11)	ELISA	OD (405 nm)>0.27^h^	No	–	+		NBA	0.5
Dazzi et al. (12)	ELISA	OD (450 nm)>3SD above mean OD of three blanks^d^	No	–	+		NBA	NR
Mondorf et al. (41)	ELISA	OD>3SD above mean OD of inhibitor negative samples (0.278)^d^	No	–	–		mBA	0.5
Boylan et al. (42)	FLI	>2SD above mean MFI HC^d^	No	–	–		mNBA	0.6
Butenas et al. (43)	MFLI	0.001 nM^g^	No	+	–		BA and NBA	0.4
Zakarija et al. (44)	FLI	>5.0 MFIU^i^	No	+	–		NBA	0.5
Krudysz-Amblo et al. (13)	FLI	>5.0 MFIU^i^	No	+	–		NBA	1.0
Clere et al. (45)	X-MAP	RAR ratio > 1^j^	No	–	–		BA	NR
Lebreton et al. (15)	X-MAP	RAR ratio > 1^j^	No	–	–		BA	0.6
Klinge et al. (23)	IP	≥4.2 IPU/mL^k^	No	+	+		NBA	0.6
Scandella et al. (46)	IP	≥4.5 IPU/mL^k^	Yes	+	+		BA and NBA	0.6 and 0.5
Irigoyen et al. (47)	FC (and ELISA)^b^	>3SD above mean OD of HC^d^	No	+	+		NBA	0.5
Shurafa and Kithier (14)	NR^c^	NR	No	–	–		NBA	NR

*The quality of the NNA assays was assessed according to the following criteria: (1) the use of positive controls and (2) competition with FVIII to establish FVIII-specificity. NNA assays were classified as high-quality (green), intermediate-quality (orange), or low-quality (red), when they met both, one or none of the quality criteria, respectively. Abbreviations: ELISA, enzyme-linked immunosorbent assay; FLI, Fluorescence based assay; IP, immunoprecipitation; X-MAP, multiplexed assay; FC, Flow cytometry; NR, not reported; OD, optical density; SD, standard deviation; HC, healthy controls; MFIU, mean fluorescence intensity unit; RAR, Relative antigenic reactivity; IPU, immunoprecipitation unit; BU, Bethesda Unit; BA, Bethesda assay; (m)NBA, (modified) Nijmegen modification of Bethesda assay*.

a *Studies used three types of recombinant FVIII products in the ELISA assays. All of these studies included one recombinant B-domain-deleted FVIII product*.

b *Study compared FC with ELISA. ELISA was not further specified in article*.

c *Name of assay was not reported, but authors briefly described the method, that included the use of monoclonal antibody-purified FVIII preparation as a source of antigen. In a previous study, this method was described in more detail (54)*.

d *In the majority of studies the cut-off for NNA positivity was calculated based on the mean OD of healthy controls plus 2 or 3SD. The number of healthy individuals varied among studies*.

e *The cut-off for positive anti-FVIII NNAs was set at 1.64 mg/mL of specific anti-FVIII IgG, corresponding to 100% specificity and 96% of sensitivity in the receiver operating characteristic curve constructed by using the results of anti-FVIII IgG measured in 107 healthy individuals and 101 patients with hemophilia A (55)*.

f *A predetermined cut-off was established for each assay using a statistical approach based on background signal levels of 160 healthy plasma donors as described in Jaki et al. (55). FVIII-specificity was only measured for high-titer antibodies (>1/80)*.

g *No further information about cut-off was given*.

h *Cut-off corresponds with an inhibitor titer > 0.5 measured with the Bethesda assay*.

i *Data were analyzed by substracting the fluorescence intensity of non-specific control ovalbulmin-coupled beads from the fluorescence intensity of specific binding of human anti-FVIII antibodies to recombinant FVIII-coupled beads. A sample was considered positive for anti-FVIII antibodies, whenever the signal of binding to recombinant FVIII beads exceeded that of binding to ovalbumin. The cut-off for positivity was set at 5.0 mean fluorence intensity units (MFIU)*.

j *Relative antigenic reactivity ratio (RAR) is the ratio between the mean fluorescence intensity (MFI) of each hemophilia A plasma and the mean MFI value of the 30 non-hemophilia plasma samples plus 3SD. The used multiplexed assay was previously described in Lavigne-Lissalde et al. (56)*.

k *The IP assay and determination of cut-off were previously described in Thompson et al. (57)*.

In nine studies both quality criteria for the NNA assay were met, including ELISA (*n* = 6), IP (*n* = 2), and FC (*n* = 1) assays (9, 10, 17, 20, 23, 33, 34, 46, 47). In the other studies, one (*n* = 10) or both (*n* = 9) quality criteria were not met. In most of these studies, FVIII-specificity had not been evaluated.

### Methodological Quality of Studies

The methodological quality assessment is summarized in Table 3. The methodological quality was high in 16/28 studies, as these studies met at least five quality criteria of the adapted JBI check list. None of the 28 included studies met all the quality criteria. Most frequently, this was because the mode of sampling was not described (*n* = 16) or the sample size was smaller than 139 (*n* = 21). Furthermore, in 27 studies, the sample coverage and response rate were unclear.

**Table 3:** JBI quality assessment.

**Source**	**Q1: Target population**	**Q2: Recruitment**	**Q3: Sample size**	**Q4: Subjects and setting**	**Q5: Sample coverage**	**Q6: Quality NNA assay**	**Q7: Measurement reliability**	**Q8: Statistical analysis**	**Q9: Response rate**
**ELISA**									
David et al. (32)	Y	Y	Y	N	U	L	Y	Y	U
Cannavo et al. (53)	Y	Y	Y	Y	Y	I	Y	Y	Y
Gangadharan et al. (17)	Y	N	N	Y	U	H	Y	Y	U
Hofbauer et al. (20)	Y	N	N	Y	U	H	Y	Y	U
Hofbauer et al. (10)	Y	N	N	Y	U	H	Y	Y	U
Klintman et al. (33)	Y	N	Y	Y	U	H	U	Y	U
Klintman et al. (34)	Y	Y	N	Y	U	H	Y	Y	U
Whelan et al. (9)	Y	Y	N	Y	U	H	Y	Y	U
Moore et al. (35)	Y	U	N	N	U	L	Y	Y	U
Lillicrap et al. (36)	Y	U	Y	N	U	I	Y	Y	U
Vincent et al. (37)	Y	N	N	Y	U	I	Y	Y	U
Towfighi et al. (16)	Y	Y	N	Y	U	L	Y	Y	U
Ling et al. (38)	Y	N	N	Y	U	I	Y	Y	U
Shetty et al. (39)	Y	N	Y	Y	U	L	Y	Y	U
Vianello et al. (40)	Y	N	N	Y	U	I	Y	Y	U
Batlle et al. (11)	Y	N	N	Y	U	I	Y	Y	U
Dazzi et al. (12)	Y	N	N	Y	U	I	Y	Y	U
Mondorf et al. (41)	Y	N	N	N	U	L	Y	Y	U
**FLUORESCENCE BASED ASSAY**									
Boylan et al. (42)	Y	Y	Y	Y	U	L	Y	Y	U
Butenas et al. (43)	Y	N	N	N	U	I	U	Y	U
Zakarija et al. (44)	Y	Y	N	Y	U	I	Y	Y	U
Krudysz-Amblo et al. (13)	Y	N	N	Y	U	I	Y	Y	U
**X-MAP**									
Clere et al. (45)	Y	N	N	Y	U	L	Y	Y	U
Lebreton et al. (15)	Y	Y	Y	Y	U	L	Y	Y	U
**IMMUNOPRECIPITATION**									
Klinge et al. (23)	Y	Y	N	Y	U	H	Y	Y	U
Scandella et al. (46)	Y	Y	N	N	U	H	Y	Y	U
**FLOW CYTOMETRY**									
Irigoyen et al. (47)	Y	N	N	Y	U	H	Y	Y	U
**NAME OF NNA ASSAY NOT REPORTED**									
Shurafa and Kithier (14)	Y	N	N	N	U	L	U	Y	U

*The questions of the JBI checklist are listed in the Supplementary Data 2. In short, the questions (Q) addressed the following issues: Q1, appropriateness of sample frame; Q2, mode of sampling; Q3, sample size ≥ 139; Q4, description of study subjects and setting; Q5, coverage of identified sample; Q6, validation of NNA assay; Q7, consistency in measurement for all participants; Q8, statistical analysis; Q9, response rate. Green = Yes (Y), Red = No (N) and Blue = Unclear (U). The colors in the column of Q6 represent the quality assessment of the NNA assay. Green = high-quality (H), Orange = intermediate-quality (I), and Red = low-quality (L)*.

### Prevalence of NNAs in All Studies

**Figure 2:**
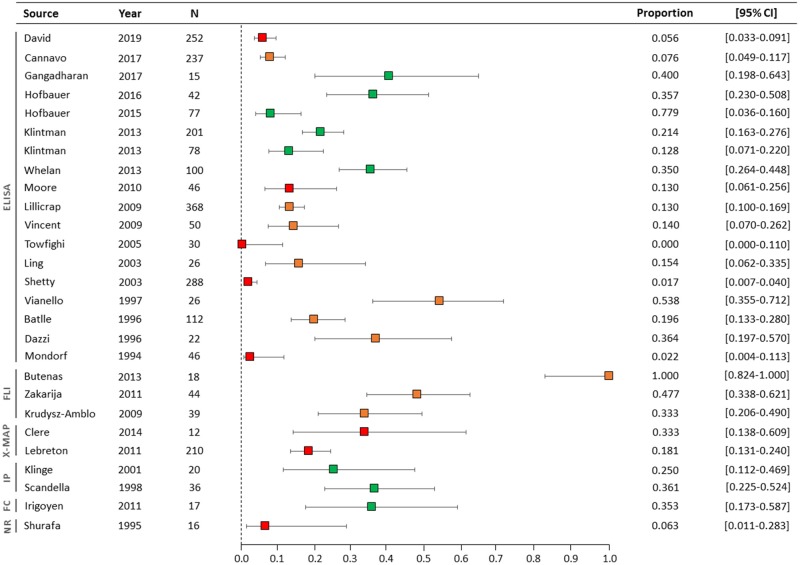
Forest plot of NNA prevalence in all studies. The NNA assay types are illustrated on the left side of the figure. The colors of the boxes represent the quality of the NNA assays: green (high-quality), orange (intermediate-quality), and red (low-quality). N, number of inhibitor-negative patients; CI, confidence interval; ELISA, enzyme-linked immunosorbent assay; FLI, Fluorescence based assay; IP, immunoprecipitation; X-MAP, multiplexed assay; FC, Flow cytometry; NR, name of assay not reported.

Overall, the prevalence of NNAs in inhibitor-negative patients ranged from 0 to 100%, with a straight unweighted average prevalence of 25% (95% CI, 4–46) (Table 4). In the nine studies with a high-quality NNA assay, the NNA prevalence ranged from 7.8 to 40% (Figure 2). Two of these studies involved previously untreated patients and NNAs were measured with ELISA and IP. Six studies were performed in previously treated patients and NNAs were detected with ELISA (*n* = 4), IP (*n* = 1), or FC (*n* = 1). One study included both previously treated and previously untreated patients and used ELISA to detect NNAs.

**Table 4:** Prevalence of NNA positive patients.

**Source**	**NNA positive patients (*n*)**	**Inhibitor negative patients (*n*)**	**Prevalence NNAs % (95% CI)**	
**ELISA**				
David et al. (32)	14	252	5.6	(3.3–9.1)
Cannavo et al. (53)	18	237	7.6^e^	(4.9–11.7)
Gangadharan et al. (17)	6	15	40.0	(19.8–64.3)
Hofbauer et al. (20)	15^a^	42	35.7^a^	(23–50.8)
Hofbauer et al. (10)	6^b^	77	7.8^b^	(3.6–16)
Klintman et al. (33)	43	201	21.4	(16.3–27.6)
Klintman et al. (34)	10	78	12.8	(7.1–22)
Whelan et al. (9)	35^c^	100^c^	35^c^	(26.4–44.8)
Moore et al. (35)	6	46	13	(6.1–25.7)
Lillicrap et al. (36)	48	368	13	(10–16.9)
Vincent et al. (37)	7	50	14	(7.0–26.2)
Towfighi et al. (16)	0*	30	0	(0–0.11)
Ling et al. (38)	4	26	15.4	(6.2–33.5)
Shetty et al. (39)	5	288	1.7	(0.7–4.0)
Vianello et al. (40)	14	26	53.8	(35.5–71.2)
Batlle et al. (11)	22	112	19.6	(13.3–28)
Dazzi et al. (12)	8	22	36.4	(19.7–57)
Mondorf et al. (41)	1	46	2.2	(0.4–11.3)
**FLUORESCENCE BASED ASSAY**				
Boylan et al. (42)	NR**	295	NR	NR
Butenas et al. (43)	18	18	100	(82.4–100)
Zakarija et al. (44)	21	44	47.7	(33.8–62.1)
Krudysz-Amblo et al. (13)	13	39	33.3	(20.6–49)
**X-MAP**				
Clere et al. (45)	4	12	33.3	(13.8–60.9)
Lebreton et al. (15)	38	210	18.1^e^	(13.1–24.0)
**IMMUNOPRECIPITATION**				
Klinge et al. (23)	5	20	25	(11.2–46.9)
Scandella et al. (46)	13	36	36.1	(22.5–52.4)
**FLOW CYTOMETRY**				
Irigoyen et al. (47)	6^d^	17	35.3	(17.3–58.7)
**NAME OF NNA ASSAY NOT REPORTED**				
Shurafa and Kithier (14)	1	16	6.3	(1.1–28.3)

CI, confidence interval; NR, not reported.^**^Study only reports the prevalence of IgG subclasses.

a *Number and prevalence of NNAs detected at lowest cut-off are shown. High-titer NNAs (cut-off: 1/80) were all of the IgG isotype (n = 9; prevalence 21.4%)*.

b *Number and prevalence of NNAs detected at lowest cut-off are shown. The overall number and prevalence of high-titer NNAs (cut-off: 1/80): 4 and 5.2%, respectively*.

c *The total group of inhibitor-negative patients was divided into two subgroups: patients without an inhibitor in the past (n = 77) and patients with an inhibitor in the past (n = 23). The overall prevalence of NNAs in these subgroups were: 34 (95% CI, 24–45) and 39 (95% CI, 22–59), respectively*.

d *4/17 inhibitor-negative patients were NNA-positive using the FC assay; 2 additional inhibitor-negative but NNA-positive patients were detected with ELISA*.

e *Confidence intervals were reported in article. The other prevalence were calculated using the Wilson method in Epitools (http://epitools.ausvet.com.au)*.

Table 5 summarizes the results of studies in which prevalence of FVIII-specific IgG subclasses or of FVIII-specific IgA or IgM isotypes were reported. In the six studies with IgG subclasses, IgG1 was the most prevalent with the prevalence ranging up to 40% (95% CI, 19.8–64.3%). NNAs of the IgG4 subclass were the least prevalent (range: 0–6.2%).

**Table 5:** Prevalence of FVIII-specific Ig isotypes and IgG subclasses.

**Source**	**Inhibitor negative patients (*n*)**	**IgA *n***	**Prevalence IgA % (95% CI)**	**IgM *n***	**Prevalence IgM % (95% CI)**	**IgG *n***	**Prevalence IgG % (95% CI)**	**IgG1 *n***	**Prevalence IgG1 % (95% CI)**	**IgG2 *n***	**Prevalence IgG2 % (95% CI)**	**IgG3 *n***	**Prevalence IgG3 % (95% CI)**	**IgG4 *n***	**Prevalence IgG4 % (95% CI)**
**ELISA**															
Gangadharan et al. (17)	15	0	0 (0–20.4)	NR	NR	6	40.0 (19.8–64.3)	6	40.0 (19.8–64.3)	0	0 (0–20.4)	0	0 (0–20.4)	0	0 (0–20.4)
Hofbauer et al. (20)	42	0	0 (0–8.4)	NR	NR	15	35.7 (23–50.8)	11	26.2 (15.3–41.1)	2	4.8 (1.3–15.8)	7	16.7 (8.3–30.6)	2	4.8 (1.3–15.8)
Hofbauer et al. (10)	77	1	1.3 (0.2–7)	1	1.3 (0.2–7)	4	5.2 (2.0–12.6)	3	3.9 (1.3–10.8)	0	0 (0–4.8)	1	1.3 (0.2–7)	0	0 (0–4.8)
Whelan et al. (9)	100	3	3 (1.0–8.5)	4	2 (0.5–7.0)	NR	NR	22	22 (15–31.1)	3	3 (1.0–8.5)	11	11 (6.3–18.6)	0	0 (0–3.7)
Towfighi et al. (16)	30	0	0 (0–1.1)	3	10 (3.5–25.6)	2	6.7 (1.8–21.3)	NR	NR	NR	NR	NR	NR	NR	NR
Batlle et al. (11)	112	0	0 (0–3.3)	NR	NR	22	19.6 (13.3–28)	NR	NR	NR	NR	NR	NR	NR	NR
**FLUORESCENCE BASED ASSAY**															
Boylan et al. (42)	295	NR	NR	NR	NR	NR	NR	69	23.3 (18.9–28.5)	26	8.9 (6.1–12.6)	9	3 (1.6–5.7)	18	6 (3.9–9.4)
**NAME OF NNA ASSAY NOT REPORTED**															
Shurafa and Kithier (14)	16	NR	NR	NR	NR	1	6.2 (1.1–28.3)	0	0 (0–19.4)	0	0 (0–19.4)	0	0 (0–19.4)	1	6.2 (1.1–28.3)

*Summary of results of studies reporting prevalences of FVIII-specific IgG subclasses or of FVIII-specific IgA or IgM isotypes. Several samples contained different populations of antibodies. Therefore, the sum of the prevalence of the individual isotypes and subclasses may be more than 100%. CI, confidence interval; NR, not reported*.

### Pooled Prevalence of NNAs in High-Quality Studies

**Figure 3:**
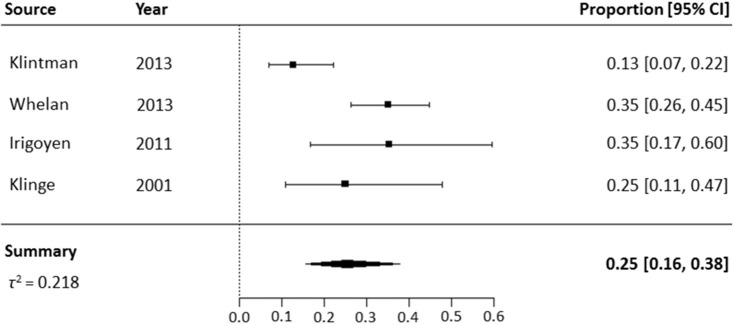
Meta-analysis of NNA prevalence in high-quality studies including previously treated patients.

Four high-quality studies that only included previously treated patients, were included in the meta-analysis of NNA prevalence (Figure 3) (9, 23, 34, 47). The NNA prevalence in these four studies ranged from 13 to 35%. The pooled NNA prevalence was 25% (95% CI 16–38%). The high-quality studies of Hofbauer et al. were not included in the meta-analysis, due to probable overlap in patient cohorts with the study of Whelan et al. (9, 10, 20). The latter study was included, as it included the largest number of patients.

### Determinants for NNA Presence

In the four high-quality studies, the majority of patients (199/215) had severe hemophilia A. In two studies reporting on inhibitor history, 27 of 178 patients had had an inhibitor in the past (9, 34). NNA prevalence was higher i.e., 24% (95% CI, 18–31%) in patients with a negative inhibitor history vs. 33% (95% CI, 19–52%) in patients with a positive inhibitor history, who had all been successfully treated with ITI.

### Incidence of NNAs

Only one study reported on the incidence of NNAs (17). In this study, 15 previously untreated patients were followed during the first 50 exposure days to treatment with rFVIII. Six of the 15 patients developed NNAs, all of IgG1 subclass with low apparent affinity, detected on at least 2 time points (NNA incidence rate: 0.01 per person-exposure day). In one of the six patients, the low-affinity IgG1 NNA was later accompanied by non-neutralizing high-affinity IgG1 NNA. The other 5 patients did not develop high-affinity NNAs and switching to other IgG subclasses was not observed.

### Association Between NNA-Status and Future Inhibitor Development

One study evaluated the incidence of inhibitor development in patients who were NNA-positive and NNA-negative at baseline before any FVIII treatment (18). In this study, 237 previously untreated patients were followed for 50 exposure days to FVIII or 3 years, whichever came first. Patients with NNAs at baseline had an 83% higher risk of inhibitor development than patients without NNAs (hazard ratio, 1.83; 95% CI 0.84–3.99). The cumulative incidence of inhibitor development was 45.4% (95% CI, 19.5–71.3%) in NNA-positive patients and 34.0% (95% CI, 27.1–40.9%) in NNA-negative patients.

### Data Evaluation

#### Small Study Data Trends

To explore the potential presence of small study data trends, the forest plot was arranged by study sample size. Asymmetry in the forest plot could be identified, due to relatively high NNA prevalences in studies with small sample sizes (Supplementary Figure 1).

## Discussion

### Summary of Results

In this systematic review, we summarized the data of 2,723 inhibitor-negative patients with hemophilia A from 28 studies to estimate the prevalence and incidence of NNAs. We found a large variety in reported NNA prevalences, ranging from 0 to 100%. In the subset of high-quality studies that included previously treated patients, the pooled NNA prevalence was 25% (95% CI, 16–38%). IgG1 was the most prevalent NNA isotype. The incidence of NNAs in inhibitor-negative patients was only given in one paper.

### Strengths and Limitations

This study is, to our knowledge, the first comprehensive systematic overview of NNA prevalence and incidence available to date. The strengths of our study were the systematic search of the literature and the extensive quality assessment of included studies, appraising the quality of both the study methodology and the NNA assay. Studies that used high-quality NNA assays and involved only previously treated patients were subsequently included in a meta-analysis, in order to provide a more reliable estimate of NNA prevalence in this subset of patients.

However, our study had several limitations. A limited number of studies reporting on the NNA prevalence was identified, including a significant number with methodological weaknesses. NNA measurement has not yet been frequently included in clinical and translational studies, because knowledge on the clinical significance of NNAs is still limited. Another limitation was the significant study heterogeneity regarding study and patient characteristics and type and quality of NNA assays. Consequently, we could only include four high-quality studies on previously treated patients in the meta-analysis, limiting the precision of the pooled estimate. Furthermore, various studies used different methods to determine cut-off values of NNA positivity. Depending on the cut-off definition, this may have led to misclassification of NNA status and over- or underestimation of the NNA prevalence. Also, the majority of studies were conducted in patients with severe hemophilia A, which limits the generalizability of the results to patients with moderate or mild hemophilia. Therefore, further research among patients with non-severe hemophilia is needed.

Our systematic review yielded only limited insight on the NNA incidence, as only one study reported on this. Furthermore, no studies on NNA occurrence in hemophilia B were identified.

### NNA Assays and Cut-Off Values

When evaluating only studies that used a high-quality NNA assay, there was more consistency in NNA prevalence. In studies that reported more extreme NNA prevalences, the quality assessment of the NNA assay was intermediate or low. The prevalence of 0% (95% IC, 0–11%) reported by one study was probably caused by the fact that this study used different cut-off values for each Ig isotype, as NNAs of IgG and IgM isotype were indeed detected in 2 and 3 patients, respectively (16). The very high prevalence of NNAs (100%, 95% CI 82.4–100%) reported by another study may have resulted from lack of evaluating FVIII-specificity, since competition with FVIII was not performed as part of the assay (43).

Use of the validated ELISA-based assay may be considered in clinical practice, because this assay meets all quality criteria and also because costs and processing time are acceptable (9).

### Determinants for NNA Presence

Several patient- and treatment related determinants for anti-FVIII inhibitor development have been described in the literature, including hemophilia severity, mutation type, and FVIII treatment (product type and intensity) (2–4, 48, 58, 59). Based on recent reports, we hypothesize that the FVIII immune response is a continuum between non-neutralizing antibodies and neutralizing antibodies and therefore the determinants of both may be similar (10, 18).

We were not able to analyze the association between hemophilia severity and the presence of NNAs due to the low number of moderate and mild patients included in the four high-quality studies. A recent study in 210 patients did not demonstrate an association between disease severity and the presence of NNAs (15).

In patients with a negative inhibitor history NNA prevalence was 24 vs. 33% in patients with a positive inhibitor history successfully treated with ITI. As there were only 2 studies that reported on inhibitor history, including a relatively low number of patients, many other study or patient characteristics might explain this observed difference in NNA prevalence (9, 33). Therefore, meta-regression analysis was not performed (60).

It is not known whether the preexisting NNAs persist after inhibitor eradication, or whether ITI itself induces new NNA formation. In one study, it has been suggested that ITI changes the subclass distribution of NNAs. In high-titer inhibitor patients undergoing ITI, a rise in the contribution of anti-FVIII IgG4 was demonstrated, independent of changes in inhibitor titer (61). Further study is needed to evaluate the association between NNA characteristics and ITI outcome and to determine if NNA presence after ITI is associated with inhibitor recurrence.

### NNAs in Healthy Subjects

In this systematic review, 9 studies also reported on NNA prevalence in healthy subjects (*n* = 2,010, NNA prevalence IQR 1.14–17%). Data are summarized in Supplementary Table 2.

The clinical significance of low-affinity NNAs in healthy individuals is incompletely understood. Previous reports indicate that low-affinity self-reactive antibodies may have a role in regulating the immune hemostasis (62, 63). In line with this, FVIII-specific NNAs in healthy individuals are hypothesized to be involved in the maintenance of peripheral immune tolerance toward FVIII (9, 10).

### Clinical Implications

Many questions remain regarding the epitope specificity, FVIII binding affinity and clinical significance of NNAs. Previous studies in patients with hemophilia as well as healthy subjects have found NNAs mostly directed against epitopes on A1, A3, and B domains of the FVIII molecule (11, 64, 65). Furthermore, Lebreton et al. demonstrated a clear immune-dominance of the complete heavy chain (A1, A2, and B-domains) in the epitope profile of NNAs, independent of hemophilia severity (15). The exact NNA epitopes remain, however, elusive and need to be characterized in future studies.

The possible effect of infused FVIII on pharmacokinetic parameters remains to be fully elucidated. Dazzi et al. demonstrated an increase in clearance rates of infused FVIII concentrate in three of 22 NNA-positive patients with negative Bethesda assays (12). This finding was supported by Hofbauer et al. who reported that high-titer NNAs modulate FVIII half-life, independent of VWF antigen level and age (20). The NNA presence was not associated with a reduced FVIII *in vivo* recovery in these inhibitor-negative patients, which is in line with two previous reports (20, 66, 67). If further studies confirm the effect of NNAs on FVIII half-life, the screening for NNAs may be considered to guide pharmacokinetic measurements.

It has been hypothesized that NNAs could serve as biomarkers for future inhibitor development. The presence of NNAs at baseline was recently demonstrated to confer an increased risk of inhibitor development (hazard ratio, 1.83; 95% CI 0.84–3.99) (18). This observation is supported by the presence of high-affinity IgG1 and IgG4 NNAs, that could be detected in an inhibitor-positive patient, in samples taken 1.5 years before the inhibitor appeared (10). It has been postulated that the affinity of NNAs could provide information on the underlying regulatory pathways involved in their generation. Hence, high-affinity NNAs of the IgG or IgA isotype are thought to be produced by long-lived plasma cells, originating from follicular differentiation pathways in germinal centers (68, 69). In line with this, Hofbauer and colleagues have suggested that NNA affinity is of more importance than NNA titers when considering the risk for inhibitor development, because even low titers of high-affinity IgG4 might indicate an evolving inhibitor (10). Adequately powered clinical studies and strict NNA monitoring are required to investigate whether high-affinity NNAs might provide an opportunity to predict and eventually prevent inhibitor development.

## Conclusion

We found a wide range of NNA prevalences in patients with hemophilia A, which resulted from considerable heterogeneity in study design with regard to disease-specific patient characteristics and type of assays used to detect NNAs. The pooled NNA prevalence was 25% in high-quality studies that included only previously treated patients and performed high-quality NNA assays. As NNA incidence was only reported in one study, more longitudinally designed studies are needed to better assess the incidence of NNAs and to further elucidate the clinical significance of these antibodies.

## Data Availability Statement

The datasets analyzed for this study are available from the corresponding author (S. C. Gouw).

## Author Contributions

AA, MB, SH, and SG: design of study. AA, MB, and SH: collection of data. AA, SH, and SG: statistical analysis. AA and JV: quality assessment NNA assays. AA, SG, and KF: redaction of manuscript. AA, MB, SH, FR, JB, JV, KF, and SG: critical review of manuscript.

## Conflict of Interest

JB is consultant for Bayer. JV is an inventor on FVIII-related patients and has received research funding from Novo Nordisk and has acted as an advisor for Biotest. The institution of KF has received unrestricted research grants from CSL Behring and Novo Nordisk and consultancy fees from Grifols, Takeda, Novo Nordisk and Roche. The remaining authors declare that the research was conducted in the absence of any commercial or financial relationships that could be construed as a potential conflict of interest.
