# Supplementary material for: Prevalence and Incidence of Non-neutralizing Antibodies in Congenital Hemophilia A— A Systematic Review and Meta-Analysis
Source: Front Immunol. 2020 May 7;11:563. doi: 10.3389/fimmu.2020.00563 (PMC7221178; doi:10.3389/fimmu.2020.00563)
Supplement: Supplementary file 1 [file Data_Sheet_1.pdf]

frontiers

Supplementary Material

1 Supplementary Data

1.1 Supplementary Data 1. Literature search

MEDLINE:

("Hemophilia A"[Mesh] OR "Hemophilia A"[tw] OR "Haemophilia A"[tw] OR "Factor VIII"[tw] OR "Factor 8"[tw] OR "FVIII"[tw] OR "Factor VIII"[Mesh:NoExp] OR ”H?emophilia B”[Mesh] OR ”H?emophilia B”[tw] OR ”Factor 9”[tw] OR ”Factor IX”[tw] OR ”Factor IX”[Mesh:NOExp] OR ”H?emophilia”[tw]) AND ("nonneutralizing"[tw] OR "non-neutralizing"[tw] OR "nonneutralising"[tw] OR "non-neutralising"[tw] OR "Antibodies, Neutralizing"[Mesh] OR (("neutralising"[tw] OR "neutralizing"[tw] OR "inhibitory"[tw]) AND ("Antibodies"[Mesh:NoExp] OR "antibodies"[tw] OR "abs"[ti] OR "antibody"[tw])))

Number of articles: 992 on 12-07-2018; 64 extra publications on 11-07-2019.

The other databases (Embase, WOS and Cochraine) were searched using the same search terms.

1.2 Supplementary Data 2. Adapted JBI checklist

JBI Critical Appraisal Checklist for Studies Reporting Prevalence Data

Reviewer____________________________________Date___________________________________

Author______________________________________Year________Record Number___________

	Yes	No	Unclear	Not applicable
**1. Was the sample frame appropriate to address the target population?**<br>• Yes: if the target population were INH- haemophilia patients<br>• No: if acquired haemophilia, INH+ haemophilia or other population (healthy donors) were studied	☐	☐	☐	☐
**2. Were study participants sampled in an appropriate way?**<br>• Yes: if random sample or consecutive	☐	☐	☐	☐
**3. Was the sample size adequate?**<br>• Yes: if sample size≥139 (expected frequency 10%; precision 5%)<br>• No: if sample size less than 139	☐	☐	☐	☐
**4. Were the study subjects and the setting described in detail?**<br>• Patients<br>Yes: if demographic data including number of participants, age, exposure days were clearly described<br>• Assay<br>Yes: if the method of antibody detection were described explicitly<br>• Cut-off<br>Yes: if the cut-off of antibody detection was mentioned	☐	☐	☐	☐

5. **Was the data analysis conducted with sufficient coverage of the identified sample?**

    - PTP/PUP ☐ ☐ ☐ ☐
    - Age
    - severity

6. **Were valid methods used for the identification of the condition?**

    - Yes: if NNA assay used positive controls as an internal standard and if FVIII specificity was measured by means of a competitive assay. ☐ ☐ ☐ ☐

7. **Was the condition measured in a standard, reliable way for all participants?**

    - Yes: if the same test with the same cut-off point was used for all participants and Sample drawn in absence of clotting factor infusions (wash out period taken) ☐ ☐ ☐ ☐
    - Unclear: if not clearly stated whether all participants were assessed similarly

8. **Was there appropriate statistical analysis?**

    - Yes: if numerator and denominator and Confidence interval for percentages were described ☐ ☐ ☐ ☐

9. **Was the response rate adequate, and if not, was the low response rate managed appropriately?**

    - Unclear for all as we do not now the response rate in the studies ☐ ☐ ☐ ☐

Overall appraisal: Include ☐ Exclude ☐ Seek further info ☐

Comments (Including reason for exclusion)

---

2 Supplementary Figures and Tables

2.1 Supplementary Figures

2.1.1 Supplementary Figure 1. Forest plot of NNA prevalence sorted by study sample size

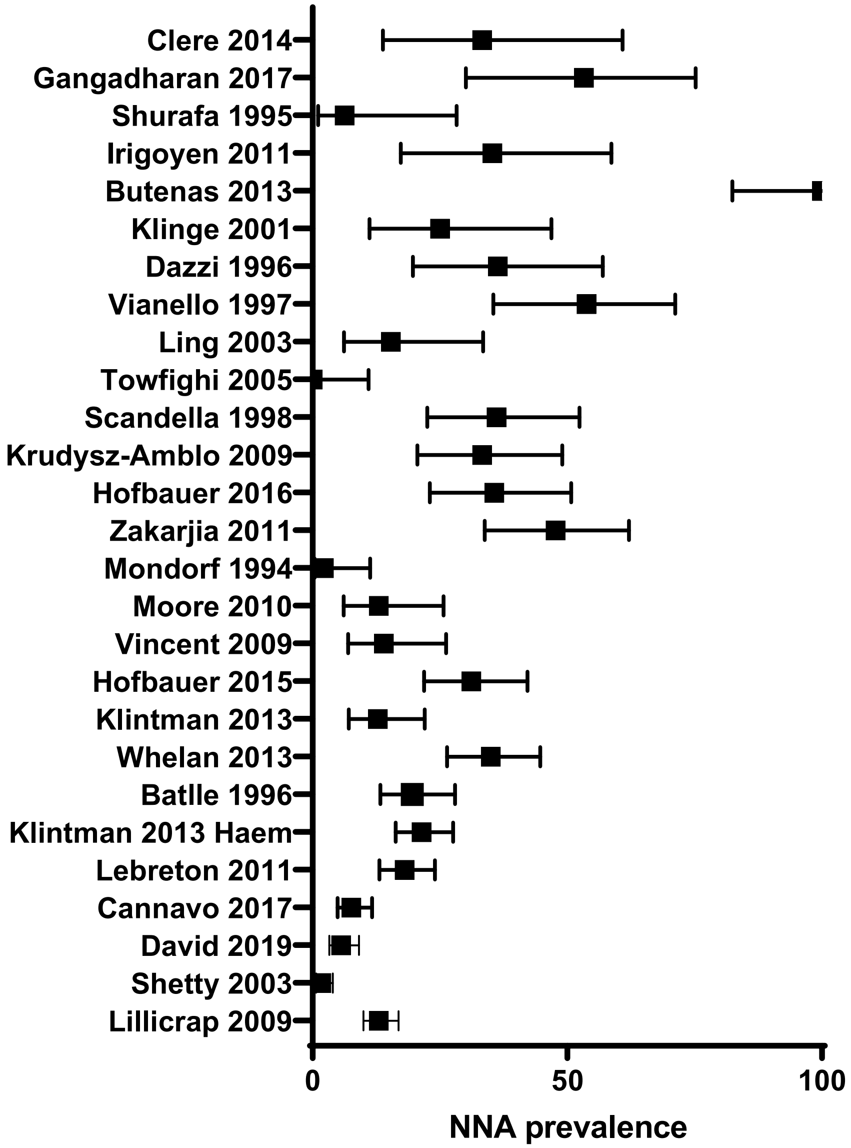

Supplementary Figure 1. Forest plot of NNA prevalences arranged by study sample size. Asymmetry in the forest plot could be identified, due to relatively high NNA prevalences in studies with small sample sizes.

2.2 Supplementary Tables

2.2.1 Supplementary Table 1. Excluded studies after further inspection

Source	Year	Country	Design	Included study population	*N* total	*N* Inhibitor negative	NNA assay	Inhibitor assay	Reason for exclusion
**Batty**	2015	UK	CS	HA, all severities, with and without inhibitor and AHA patients.	225	NR	ELISA[a]	NBA and mNBA	Calculation of prevalence was not possible, due to multiple samples. Therefore, some patients may have had samples that were Bethesda and/or ELISA positive. This was verified with the author of original study.
**Riddell***	2013	NR	CS	HA, all severities and AHA patients.	109	NR	ELISA[b]	NBA	Calculation of prevalence was not possible, because the total number of inhibitor-negative patients also included patients with AHA (number not reported). Therefore the total number of inhibitor-negative patients with hemophilia A, was not known. The second reason for exclusion was inconsistency in reported patient numbers.

Abbreviations: CS, cross-sectional; NR, not reported.

*Conference abstract. [a] In Batty *et al.* NNAs were detected, when the optic density > kit controls (KC). The KC was derived from human serum containing antibodies to human FVIII. The KC is lot specific, defined by dilution studies of a known positive sample and is tested by the manufacturer to ensure that the threshold results in the expected reportable results in over 90 test samples (positive/negative Bethesda activity).[b] In Riddell *et al.* the cutoff for the ELISA was not reported.

References:

1. Batty P, Moore GW, Platton S, et al. Diagnostic accuracy study of a factor VIII ELISA for detection of factor VIII antibodies in congenital and acquired haemophilia A. *Thromb Haemost.* (2015). doi:10.1160/TH14-12-1062
2. Riddell A, Pickering WM, Lawler P, et al. Comparison of a ELISA FVIII inhibitor assay with the Nijmegen Modified Bethesda assay in patients with inherited and acquired haemophilia A. *J Thromb Haemost.* (2013) 2:935

2.2.2 Supplementary Table 2. Prevalence of NNAs in healthy subjects

Source	Healthy subjects		
	N NNA-positive	N HS	NNA prevalence % (95% CI)
**Hofbauer**	17[a]	634	2.7 (1.7 - 4.3)
**Whelan**	NR	600	19 (16 - 22)
**Vincent**	0	44	0 (0 - 8.0)
**Shetty**	0	31	0 (0 - 1.1)
**Batlle**	8	53	15.1 (7.9 - 27.1)
**Dazzi**	4	20	20 (8.1 - 41.6)
**Mondorf**	25	460	5.4 (3.7 - 7.9)
**Krudysz-Amblo**	4	150	2.7 (1.0 - 0.07)
**Shurafa**	2	18	11 (3.1 - 32.8)

Abbreviations: HS, Healthy subjects.

[a] These NNA-positive HCs had a high-titer FVIII NNA.

References:

1. Hofbauer CJ, Whelan SFJ, Hirschler M, et al. Af fi nity of FVIII-speci fi c antibodies reveals major differences between neutralizing and nonneutralizing antibodies in humans. 2015;125(7):1180-1189. doi:10.1182/blood-2014-09-598268.
2. Whelan SFJ, Hofbauer CJ, Horling FM, et al. healthy individuals and in different cohorts of hemophilia A patients Distinct characteristics of antibody responses against factor VIII in healthy individuals and in different cohorts of hemophilia A patients. 2013;121(6):1039-1048. doi:10.1182/blood-2012-07-444877
3. Vincent AM, Lillicrap D, Boulanger A, et al. Non-neutralizing anti-FVIII antibodies: Different binding specificity to different recombinant FVIII concentrates. Haemophilia. 2009. doi:10.1111/j.1365-2516.2008.01909.x
4. Shetty S, Ghosh K, Mohanty D. An ELISA assay for the detection of factor VIII antibodies - Comparison with the conventional Bethesda assay in a large cohort of haemophilia samples. *Acta Haematol*. 2003. doi:10.1159/000067272
5. Batlle J, Gómez E, Rendal E, et al. Antibodies to factor VIII in plasma of patients with hemophilia A and normal subjects. *Ann Hematol*. 1996. doi:10.1007/s002770050179
6. Dazzi F, Tison T, Vianello F, et al. High incidence of anti-FVIII antibodies against non-coagulant epitopes in haemophilia A patients: A possible role for the half-life of transfused FVIII. *Br J Haematol*. 1996. doi:10.1046/j.1365-2141.1996.d01-1705.x
7. Mondorf W, Klinge J, Luban NLC, Bray G, Saenko E, Scandella D. Low factor VIII recovery in haemophilia A patients without inhibitor titre is not due to the presence of anti-factor VIII antibodies undetectable by the Bethesda assay. *Haemophilia*. 2001. doi:10.1046/j.1365-2516.2001.00463.x
8. Krudysz-Amblo J, Parhami-Seren B, Butenas S, et al. Quantitation of anti-factor VIII antibodies in human plasma. *Blood*. 2009. doi:10.1182/blood-2008-08-174987
9. Shurafa M, Kithier K. IgG antibodies against factor VIII in normal individuals. *J Thromb Thrombolysis*. 1995. doi:10.1007/BF01064378
